# Adaptive Gaze Behavior and Decision Making of Penalty Corner Strikers in Field Hockey

**DOI:** 10.3389/fpsyg.2021.674511

**Published:** 2021-08-02

**Authors:** Stefanie Klatt, Benjamin Noël, Alessa Schwarting, Lukas Heckmann, Frowin Fasold

**Affiliations:** ^1^Institute of Sports Science, University of Rostock, Rostock, Germany; ^2^Institute of Exercise Training and Sport Informatics, German Sport University Cologne, Cologne, Germany; ^3^School of Sport and Health Sciences, University of Brighton, Eastbourne, United Kingdom

**Keywords:** drag-flick, eye-tracking, performance, sport expertise, tactical decision

## Abstract

In recent years, studies have increasingly dealt with the interaction of gaze behavior and decision making of team sports athletes. However, there is still a variety of important game situations, for example, in the case of penalty corners in field hockey, in which this interaction has not been investigated in detail yet. Penalty corners present a meaningful goal scoring opportunity by providing a relatively free shot. This paper considers two studies. The first study investigated a possible connection between the gaze behavior and the quality of decisions of experienced field hockey players and evaluated the level of success of different gaze strategies. A preliminary study (Study 1) was designed as a survey questionnaire with the aim of preparing for the main study by obtaining subjective assessments of the individual gaze behavior and decision making of professional athletes. In the second and the main study (Study 2), the gaze behavior of experienced field hockey players was recorded using mobile eye-tracking systems to analyze different strategical approaches in associated gaze behavior and decision making. Study 1 showed that players consider reacting to the defenders' behavior during a penalty corner a promising avenue for improving success at penalty corner attempts. It also indicated that such defense-dependent strategies are currently only rarely employed. Study 2 demonstrated how gaze behavior differs between different strategical approaches of the offense. It was shown that the gaze direction on the ball, the stopper, and the goal area is important to allow for a more optimal adaptation to the tactical behavior of defense. It can be concluded that adaptive decision making (i.e., choosing which variation will be carried out just after the “injection” of the ball) seems promising but requires further training to improve the success rate of penalty corner.

Nowadays, a lot of games are decided by penalty corners in field hockey. For example, during the 2018 World Cup, the four group winners scored 38% of their goals through penalty corners in the preliminary rounds, while about a third of the goals scored against the four teams eliminated in the preliminary rounds were scored through penalty corners. Surprisingly however, there is currently little research on arguably the most important action in field hockey (for a study on indoor field hockey see Vinson et al., 2013). Only recently some studies have mainly addressed the role of the goalkeeper in defending penalty corners (Morris-Binelli et al., 2020, 2021). Our study addresses this game situation and tries to scrutinize if more (online) adaptation on offense can be considered a promising future development of behavior during penalty corner by questioning (Study 1) and testing (Study 2) expert field hockey players.

During a field hockey game, the penalty corner (or short corner) is considered a highly complex strategic part (Laird and Sutherland, 2003). It is awarded for fouls committed by the defending team within the goal-scoring circle or for serious fouls outside of this circle (intentional rule violations). Before the penalty corner is carried out, four defending players and the goalkeeper position themselves behind the backline, either next to or inside the goal. All other defending players must be beyond the centerline. Any number of players of the attacking team can position themselves outside the circle with sticks, hand, and feet not touching the ground inside the circle. One player from the attacking team, the “injector,” passes the ball from the backline at least 10 meters from the goalpost, usually to a player stopping the ball (stopper) for another player (striker) who either shoots the ball directly or passes it to yet another player (who may or may not take a shot). After the ball has been played by the injector, the defending players and the goalkeeper are allowed to enter the circle. The stopper of the attacking team must receive the ball outside of the shooting circle or else, briefly leave the circle after receiving the ball before any other actions can be taken.

Even though it seems that straight shots at the goal by the striker promise the greatest rate of success, there will possibly be a much greater demand for various penalty corner variations that demand online strategical decision making in the next few years due to adaptations in defenders' behaviors and lower success rates (average conversion rate World Cup 2018 ≈ 20.5%, Rowe, 2019). Defensive behavior and play calling are now increasingly taking into account that on offense in most cases a straight shot is called. Various analyses of penalty corners during past international tournaments (World Cups and Olympic Games) have shown that the penalty corner effectiveness depends on the fit between strategical approaches of the offense and defense, that is, whether a direct shot or a pass is performed (cf. Vizcaya, 2015) and how defensive players run-out to prevent goal scoring. This implies that the strikers could have an advantage if they were able to recognize the defensive behavior of the opponent's team early in order to choose a more promising option for the penalty corner.

This is also done in comparable situations in other team sports as the penalty kick in soccer. In this situation, the penalty taker can choose to decide on kick direction prior to running-up the ball and stay with that decision regardless of what the goalkeeper subsequently does. This strategy is termed keeper-independent strategy (Kuhn, 1988; van der Kamp, 2006). However, strikers can also choose to employ a keeper-dependent strategy in which they decide for a temporal target but mainly wait for the goalkeeper to commit to one side only to kick to the opposite side of the goal (Kuhn, 1988; van der Kamp, 2006; Noël et al., 2014). Importantly, both strategies are associated with fundamentally different patterns of gaze behavior probably reflecting the fact that penalty takers employing a keeper-dependent strategy rely on information on the goalkeeper' behavior (Noël and van Der Kamp, 2012). Furthermore, it was shown recently that the likelihood of scoring depends on the combination of the goalkeepers' behavior and the strategic approach of penalty takers (Noël et al., 2021).

In field hockey, an approach similar to the keeper-dependent strategy would create the opportunity to adapt to the run-up behavior of the defensive players after the ball is in play in order to choose either a straight shot or another penalty corner variation (even though, in penalty corners, it is not only the striker who has to adapt, but also his/her teammates). In this regard, it is important to consider which general variations are usually played and with which defensive tactics (see also Study 1). Therefore, the following offensive and defensive tactics are basic schemes that are subject to a high degree of variability. Due to the different possible variants, the positions and routes of the players differ according to the situation and rarely follow an exactly identical structure.

In international field hockey, the direct shot at goal is the variation that is most often played. By using the drag-flick, where the ball is dragged on the shaft of the hockey stick and flung at the goal, the striker can reach high velocity of the ball (Baker et al., 2009). Furthermore, the drag-flick allows to raise the ball over 460 mm (backboard height; Ibrahim et al., 2017), which is the critical height for hits (striking by using a swinging movement of the stick toward the ball) in penalty corners. Another option that is regularly chosen is the 90° variation. Here, the striker hooks the ball into the stick head during the initial movement of the flick. Instead of releasing the ball in front of the body in the direction of the goal, the striker rotates 90° to the left to a teammate who receives the ball and shoots it at the goal. Thereby the 90° variation is an offensive tactic allowing a different striker to shoot from a closer position to the goal with slightly more time to do so. This variation belongs to the general category of “pass to the left.” For the deflection variation, the ball is brought into play by the injector, while an attacker runs into the zone between the penalty spot and the backline and lays down his/her hockey stick on the ground to deflect the ball. The striker takes a low shot at the goal using a drag-flick or a hit. The hockey stick that has been laid down by the other attacker deflects the ball either high or into another direction or both, so that the goalkeeper and the defensive players at the goal-line only have a very short time to react.

In general, two different defensive tactics are commonly chosen by the defense, both of which are initiated from the same starting formation in order not to provide the opponents with any cues for their decisions before the ball is in play. In the commonly used starting formation, three defenders stand to the right and one defender stands to the left of the goalkeeper (from the perspective of the offensive players looking at the goal)^1^. This formation is referred to as 3:1. Two options arise from this situation: *3:1 3:1 and 3:1 2:2*. In the 3:1 3:1 situation, one defender (first runner) of the block of three runs up to the striker in order to cover the right corner of the goal as soon as the ball is brought into play by the injector. Another one of the players (trailer) on this side remains positioned slightly offset behind in order to defend possible deflection variations. The remaining player on this side stays on the goal line to be able to parry the shots that cannot be prevented by the first defender. The defender to the left of the goalkeeper positions himself/herself between the penalty spot and the goal-line to be able to clear possible rebounds from the zone in front of the goalkeeper. Due to the fact that the right corner of the goal is defended directly by two players, the goalkeeper has the possibility of taking a step toward the left goalpost and of focusing more strongly on this corner of the goal. In the end position, three defenders stand to the right and one defender stands to the left of the goalkeeper (3:1).

In the *3:1 2:2 situation*, from the starting formation, the defender standing to the left of the goalkeeper runs toward the pass to the left of the striker. One defender of the block of three to the right of the goalkeeper runs up to the striker to cover the right corner of the goal. Another one of the players moves behind the goalkeeper to the left side and positions himself/herself in the zone between the penalty spot and the goal line. His/her task is to clear possible rebounds from the zone in front of the goalkeeper and defend variations. The remaining defender on the right side stays on the goal line in order to parry shots that cannot be prevented by the first defender. In the end position, two defenders are standing to the left and two to the right of the goalkeeper (2:2).

The two defense variations each prevent another possible attack strategy, respectively. By using the 3:1 3:1 the defenders are positioned closely around the penalty spot and supposed to defend possible deflection strategies. The 3:1 2:2 is supposed to defend the pass to the left (here 90° variation). Regardless of the defense variation, the drag-flick (direct shot) always represents a reasonable variant and an effective shooting technique when it comes to the penalty corner (Piñeiro et al., 2007; Rosalie et al., 2017), although, it cannot always be considered optimal because of the defenders' positioning at the goal-line. For the defense variation 3:1 the 90° variation represents an optimal counter strategy, because no defender is directly assigned to the attacker who receives the ball from the striker. Whereas, for the 2:2 variation, it represents a less appropriate counter strategy because in this case the player on the 90° position is directly defended. In contrast, the deflection variation is the optimal solution for the 2:2 variation, because of the relatively wide-open zone between the two defenders who are defending the attackers at the top of the circle and the two defenders focusing more on the zone close to the goal. While the defense is using a 3:1 variation a deflection variation seems ineffective in comparison to the 90° variation referring to the higher number of defenders around the penalty spot possibly interfere the execution of the variation.

However, in reacting on the defensive players' run-out, strikers would probably also have to deal with the same problems as penalty takers in soccer employing a keeper-dependent strategy. On the hand, they have to focus on the behavior of the defensive players but on the other hand they also have to prepare for/focus on the execution of their own actions (Noël and van Der Kamp, 2012). However, currently it is not known if and how often offensive players try to employ a “defense-dependent” strategy, but it appears that in the majority of the cases a “defense-independent” strategy is employed. It is also not known if it is possible to focus on aspects of defenders' behaviors while also preparing and coordinating self-actions given that it takes <2,000 ms from the ball being injected till the ball leaves the strikers stick. Furthermore, in penalty corners it is not only the striker who has to come to a reasonable decision in time, but also the teammates have to perceive the situation correctly to act accordingly. It is certainly possible that play callers consider these demands on the gaze behavior of players a major problem and therefore rely on a defense-independent strategy only in which every player on offense knows prior to the injection of the ball which variant (e.g., a direct shot) will be played.

Although, in general, a number of investigations of the combined gaze and decision-making behavior in high-performance sports is available (for reviews, see Kredel et al., 2017; Hüttermann et al., 2018), focusing on predominantly foveal (e.g., Noël and van Der Kamp, 2012) as well as peripheral vision (Vater et al., 2017), in field hockey, only Roth et al. (2007) have evaluated the gaze behavior of strikers so far. It was found that, as the ball is put into play, the defensive players running out of the circle had their gaze fixated by the striker in order for them to choose their action accordingly. As soon as the ball was stopped, only the ball was fixated by the striker. Thus, anticipatory processes may play a role in action selection since the strikers subsequently did not fixate on the space or the defenders anymore. Alternatively, peripheral vision can potentially be used by strikers through this process. Here, strikers may predominantly focus on the ball while monitoring other important aspects, like the stopper, peripherally (cf. Klatt and Smeeton, 2021a,b). In this way, such patterns of gaze may function as a gaze anchor (cf. Vater et al., 2016). However, because only two athletes were tested within the scope of these investigations by Roth et al. (2007) and the quality of the decision-making behavior was not considered, the meaningful relationship between gaze behavior and decision-making behavior remains unclear. Particularly this aspect, however, is of vital importance for the success or failure of penalty corners as the decision for an offensive play call that matches well with the opponent's defense strategy significantly increases the success rates of penalty corners (cf. Vinson et al., 2013).

Because of the explorative nature of this research, a questionnaire was developed in Study 1 which was designed to collect information about penalty corners from the perspective of expert players. That is, we mainly wanted to get basic information on the current state of penalty corners while also finding out how expert players think about application of a defense-dependent strategy in penalty corner situations, if they have already had some experience with these kinds of approaches and how they would describe their own gaze behavior and demands on their own gaze behavior (though reliability of self-reports on gaze behavior is limited, e.g., Kok et al., 2017; van Wermeskerken et al., 2018).

In the main study (Study 2), we scrutinized to what extent different strategical approaches are associated with different patterns of gaze and if strikers are able to distribute their allocation in a way that allows them to gain information on defenders' behavior on the one hand and focus on the execution of their own actions until the ball has reached the stopper, on the other. Furthermore, a fundamental difference between successful and less successful athletes across a range of different types of sports and sports situations is the ability to apply their visual perceptual skills in a targeted manner in order to be able to operate, anticipate, and react successfully (cf. Williams et al., 1999; Starks and Ericsson, 2003). Thus, Study 2 was also aimed at investigating decision-making behavior (decision quality: optimal vs. less appropriate) as a function of gaze behavior during employing defense-dependent and -independent strategies in the penalty corner in field hockey.

## Study 1

Study 1 was conducted as a preliminary study for Study 2. Given the explorative nature of this current research, it was important to generate subjective assessments about experts' behavior during penalty corners. In general, the following questions were answered by the participants through the questionnaire: (1) What is the importance of penalty corners during training for professional teams? (2) Which offense and defense tactics are preferable in professional teams and do they validate our initial assumptions? (3) How do experts behave during penalty corners from a tactical perspective? (4) How can defense-dependent strategies be implemented, i.e., where and when do players think one should gain information on the defenders' strategic behavior?

### Method

#### Participants

In total, 48 (31 male, 17 female) participants completed the questionnaire. About 19% of all participants actively played field hockey in the highest German league and 81% in the second highest league at the time of data collection. About 17% of the participants reported having <1 year of playing experience in the two highest German leagues, about 50% had 1–5 years, 25% 5–10 years, and 8% more than 10 years of playing experience.

#### Materials and Procedure

The questionnaire^2^ included 29 questions and was created online using the EFS Survey program (Questback GmbH, Germany). First, questions were asked about the preparation of penalty corners for the specific match in order to find out about the importance of penalty corner training in general. The survey also indicated whether the implementation of defense-dependent strategies seems promising from a player's perspective. Second, questions were asked about the theoretical preparation. Subsequently, participants answered questions concerning their (gaze) behavior *during the match*. Here the focus was on assessing individual gaze strategies and possible gaze strategies in defense-dependent approaches.

### Results

Forty-eight participants indicated that they performed a penalty corner training at least once a week at the time of the data collection. More than half of the participants reported to train for penalty corners in a second session per week in addition to their regular practice. More than 70% of the participants took part in at least one training session specifically designed to improve penalty corner performance. Almost 80% of the respondents indicated that a penalty corner training during a team training unit usually lasted 15–30 min. The duration of the additional penalty corner training was around 30–45 min for approximately half of the respondents.

Players indicated that the penalty corner drag-flick is indeed the variation they most often choose and train for, followed by the 90° and deflection variations. Almost all the participants prepared for these variations using video analyses of the players from opposing teams. They indicated that countering the defense variation 3:1 3:1 with the 90° variation is the most appropriate solution, while the efforts do not match well with the 3:1 2:2 variation. They also supported our initial assumption that the deflection variation is optimal for countering the 3:1 2:2 variation, while being largely ineffective for the 3:1 variation.

**Figure 1:**
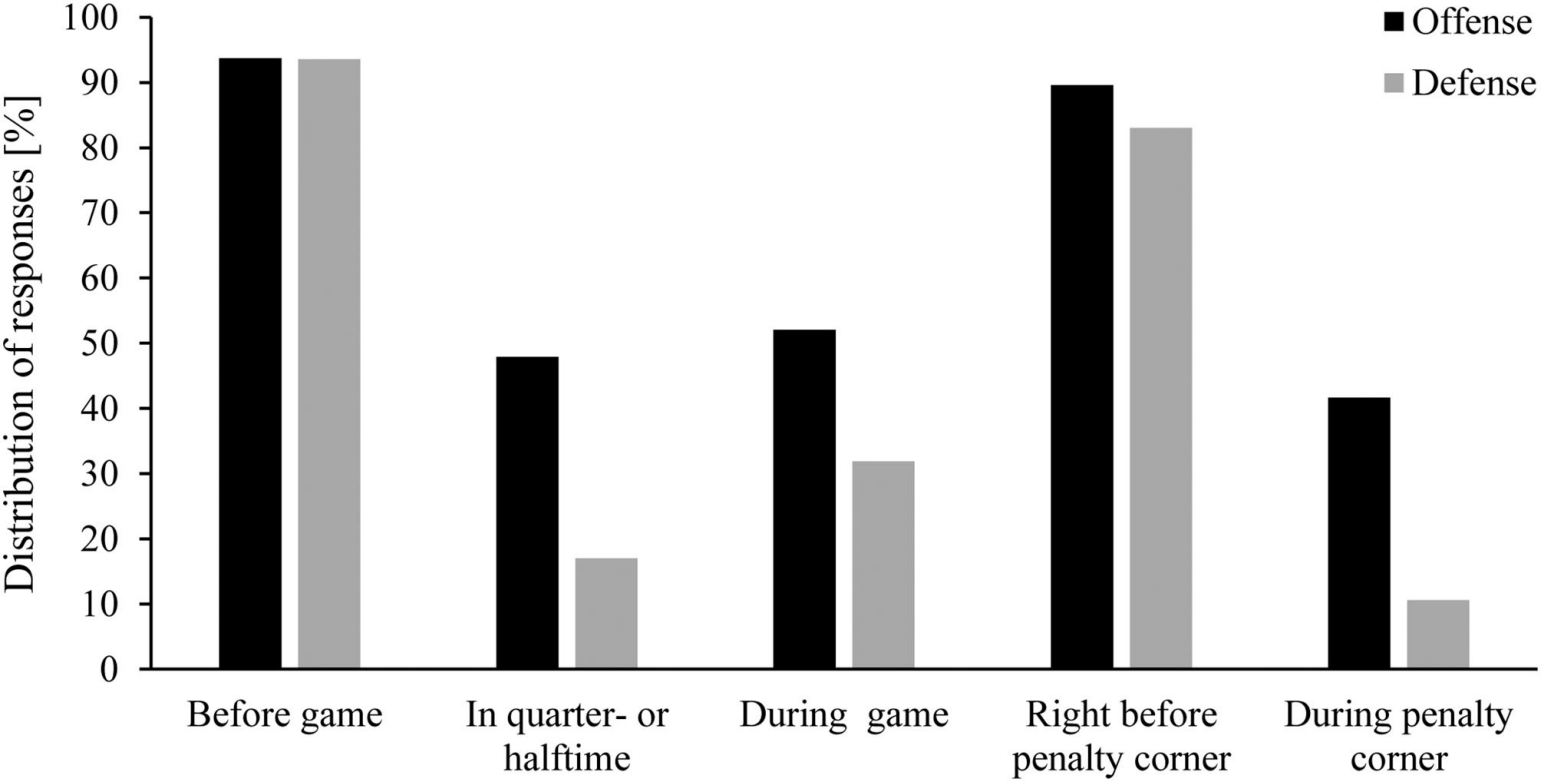
Distribution of answers to the question when a decision is made for a certain penalty corner variation during competition by offensive players and defensive players. Participants were allowed to give multiple answers.

Usually, with prior training, players decided on the variation to use either before the match or just before the penalty corner based on the knowledge of the tendencies of the opposing team. Though it was found that approximately 40% of the participants, at least once tried to adapt their strategical approach to the run-up behavior of the defensive players during the penalty corner, players also indicated that in the vast majority of the cases, they employed a defense-independent strategy (Figure 1).

**Figure 2:**
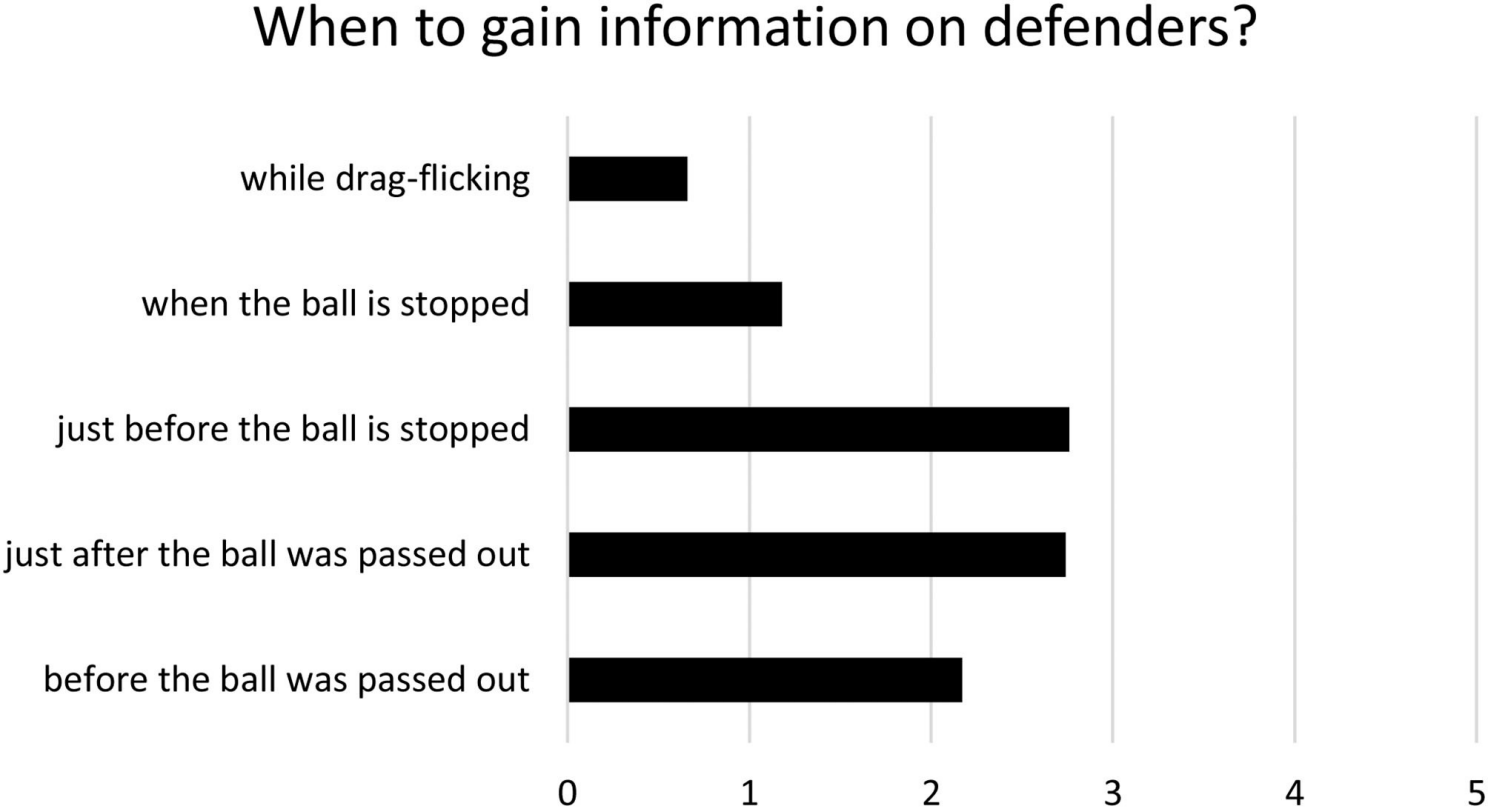
Hockey players' opinion on when it is reasonable to acquire information on the defenders' run-out behavior/formation during penalty corners on a five-point-scale (1 = not possible/reasonable at all, 5 = very well possible/reasonable) in order to react to it by choosing a well-matching offensive variant.

With regard to the individual gaze strategies of the striker, 60% of the participants indicated that they had consulted with their coach about gaze behavior before. Regarding the time when gaze could be directed at the defenders to identify their strategy, “just after the ball was injected,” and “just before the ball is stopped” were the most common responses by the participants (Figure 2). They emphasized that in these situations, but not while drag-flicking, there usually would be sufficient time to react to the run-out of the defenders, i.e., the preparation of the variation and the movement execution as a response to the strategy of the defenders. Unsurprisingly, most of the players named parts of the defense (“position trailer,” “position first runner”) or the “whole defense block” as information rich areas they would focus on to identify defensive strategies. Moreover, they named the deflection and 90° variations as the most suitable variations to react to the defenders' behavior.

### Discussion

The results of the questionnaire indicated that there are frequent and regular penalty corner trainings in the first German division in field hockey. With a total duration of 2 h of training and an average of 15–30 min of specific penalty corner training, almost a quarter of the training is used exclusively for penalty corners. Many teams perform an additional penalty corner training to improve their success rate. In order to be prepared for the opponent, penalty corners as well as the opponent's team tactics are discussed during competition preparation. Based on these discussions, the penalty corner training is adapted and the different variants are determined before the actual match or penalty corner. The results of the questionnaire also illustrate that the offense variation is chosen shortly before the penalty corner in most cases, i.e., seemingly, the defense-independent strategy is employed much more often than the defense-dependent strategy even though the participants' level of expertise was relatively high. This may help to explain why in the past years, many teams have been able to lower the success rates of very good strikers through planned and purposeful defense behavior: they were able to know what to expect and tried to employ a more promising counter strategy. If offense players more often employed defense-dependent decision making instead of the currently prevailing defense-independent decision making, it could help to select the offense strategy that fits best with the defenders' strategy. This would make it nearly impossible to prepare an effective counter strategy for defense players as well. The players themselves considered this as a promising development. They indicated that the pass to the left (90° variation) and the deflection variation are the most suitable for short-term changes during actual penalty corner attempts, supporting our initial assumptions on which areas of interest may play a more important role in defense-dependent compared to defense-independent strategy.

## Study 2

Based on the results of Study 1, and because self-reports on gaze behavior are not always reliable (cf. Kok et al., 2017; van Wermeskerken et al., 2018), Study 2 was designed as a field study with the goal of analyzing gaze and decision-making behaviors of strikers in field hockey. We aimed to scrutinize if (and how far) the gaze behavior differs between attempts in which the players know which variant they will play and attempts in which they try to counter the defending team's tactical approach (defense-dependent vs. defense-independent strategy). Thereby, the defense-independent strategy may be considered the players' natural (normally employed) strategy, based on what they reported in Study 1. In line with the information provided by the players in Study 1, we hypothesized that identifying tactical approaches of the defense requires looking longer at the goal area, especially early on (i.e., roughly until the ball reaches the stopper). This is so because later, the attention has to be directed to the stopper and the ball to guarantee optimal execution while possibly also perceiving information from other areas of interest (e.g., the stopper) peripherally (cf. Hüttermann et al., 2013; Klostermann et al., 2020). In contrast, while employing a defense-independent strategy, strikers theoretically can exclusively focus on offense related aspects of the penalty corner, such as the injecting player, stopper, and ball, early on because regardless of the defenders' run-out they would follow the same action plan anyway. So, there is a much lesser need to gain information on the defenders' behavior at that point. Furthermore, we also wanted to test if successfully identifying the defenders' tactical approach benefits from certain patterns of gaze behavior. We analyzed if and in how far gaze behavior during attempts in which players tried to identify and react to the defender's behavior differs between successful and unsuccessful trials. We expected that relatively more time will be spent looking at the seemingly more informative areas of interests (i.e., goal area and ball) in the cases the strikers made an optimal decision compared to an appropriate decision. Because of the results of Study 1 and also because several areas of interest (the injector, stopper, and goal area) are spatially far apart, we did not expect strikers to focus on areas in between until the ball had reached the stopper.

### Method

#### Participants

In total, 14 strikers (3 female, 11 male) took part in the experiment. Due to technical difficulties, the eye-tracking data of one participant could not be evaluated and had to be excluded. The average age of the participants was 21.93 years (*SD* = 3.95 years). At the time of the experiment, the participants had been active as field hockey players for 16.71 years on average (*SD* = 2.53 years). Six of the participants (3 female, 3 male) had experience in the first German Division (*M* = 3.33; *SD* = 2.34). Nine of the players indicated experience in the second German Division (*M* = 2.67; *SD* = 2.29). Two of the participants also had experience as a striker in senior national teams (5 years) and another three where part of a youth national team for 1.67 years on average (*SD* = 0.58). The experiment was carried out in accordance with the Helsinki Declaration of 1975, and the participants signed a consent form approved by the local ethics committee.

#### Materials

The gaze behavior of each participant was recorded using a mobile eye-tracking system (Pupil Labs GmbH, Berlin, Germany). A mobile eye-tracking headset connected to a mobile bundle consisting of a Motorola Moto Z2 or Z3 Play with an USBC-USBC cable was used. The two eye cameras of the eye tracker had to be configured to record the full scope of the movement of the pupil in all movement directions. The front camera of the eye-tracking system had to be adjusted so that the entire visual field of the striker was recorded (120 frames per second). The gaze information of both eyes was recorded at 200 Hz and matched with a simultaneously captured scene video recorded at 30 Hz.

The game situations were recorded by two cameras (GoPro Hero 8 black, GoPro, Sam Mateo) from behind the striker and from a lateral perspective, to be able to view and assess all movements from two different viewing positions.

#### Procedure

The testing of each participant took around 30 min including the instruction, a warmup, and the actual testing. Initially, the test setup and procedure were explained, and the eye-tracking glasses were adjusted for each participant individually. After configuring the mobile recording devices (Moto Z2 or Z3 Play with Pupil Mobile App), they were connected, and the calibration was performed prior to the start of the testing.

**Figure 3:**
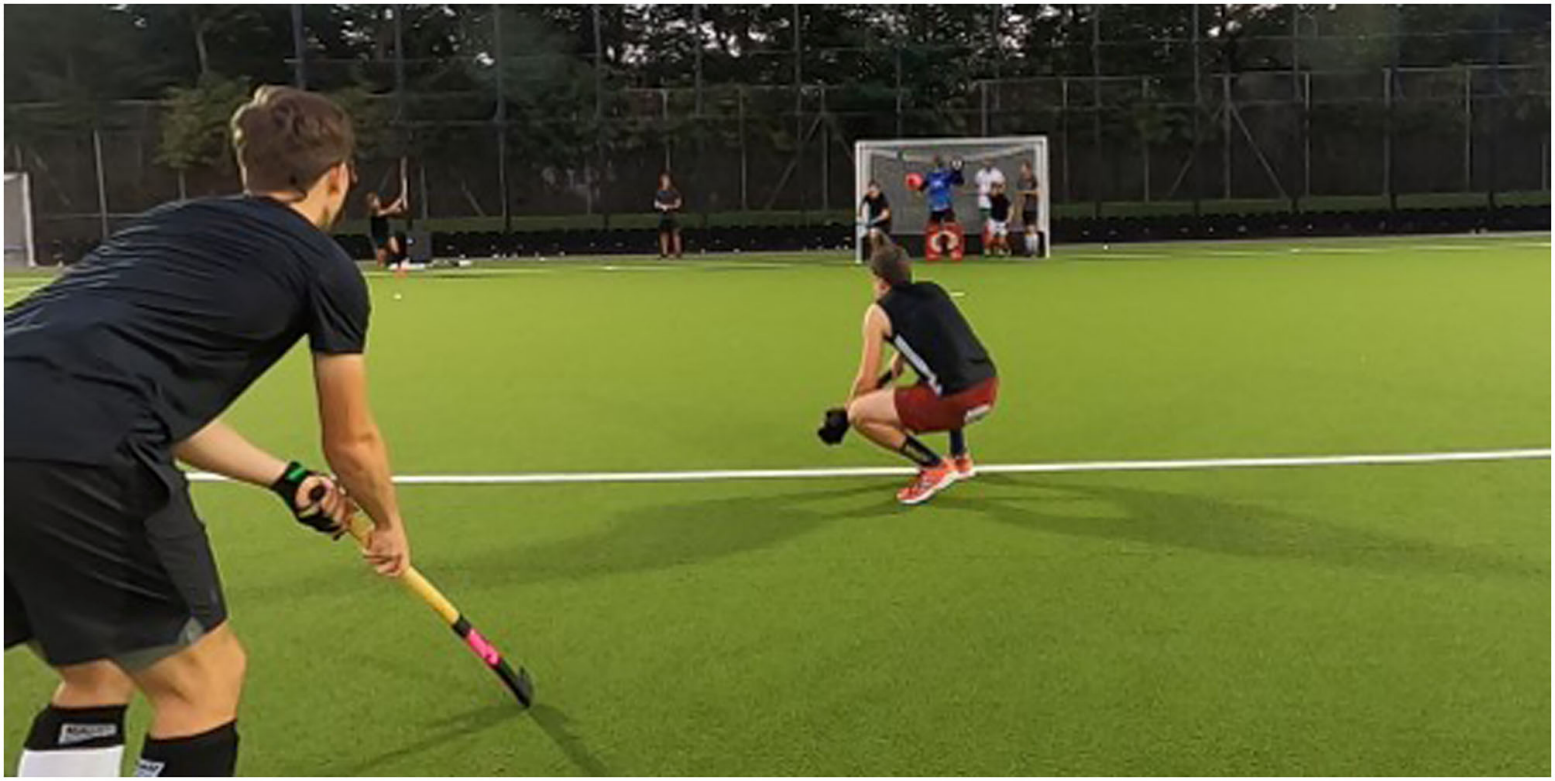
Depiction of field testing from the perspective behind the striker showing the striker (standing) and stopper of the offensive team in the foreground and the defenders and goalkeeper inside the goal before running out just after the injector (far left from the goal) has played the ball.

Each participant performed 20 penalty corners as the striker (see Figure 3). In half of these penalty corners, the penalty corner variations were given beforehand (defense-independent strategy). The combination of penalty corner variations (shot, 90°, or deflection) were chosen in random order. In the other half players were asked to react to the run-out behavior of defenders (defense-dependent strategy). Importantly, defenders' strategy was always unknown to the offense and thus had to be identified during the penalty corner attempt (see Supplementary Table 1). Each defense variation was played five times in random order (5x 3:1 3:1, 5x 3:1 2:2) and defense variations were kept the same between both conditions. The striker's task was to find an optimal solution for the situation. To this end, three solution possibilities were available: penalty corner drag-flick, 90° variation, and deflection variation but it was emphasized that only the latter two were considered optimal solution depending on the run-out behavior of the defenders whereas the direct shot (penalty corner drag-flick) was considered a fallback option (which is never completely inappropriate to use).

The experimental differentiation between the defense-dependent and defense-independent situations served to distinguish varying demands on gaze behavior. That way, both visual sources of information for the successful execution of an action (shot, pass) as well as sources of information for making correct decisions in the penalty corner situation were meant to be identified.

#### Data Analysis

First, the recordings of those cameras that recorded the individual shots were sifted through entirely. The video recordings served to identify the running behavior of the defense and, thereby, reconstruct the participants' decision-making behavior. The execution of the penalty corners was divided based on the decision quality (optimal vs. less appropriate) in this context by choosing the 90° variation as the optimal solution for 3:1 3:1 and the deflection variation as the optimal solution for 3:1 2:2. Respectively, the deflection variation was less appropriate for the 3:1 3:1 defensive variation and the 90° variation was less appropriate for the 3:1 2:2 variation.

**Figure 4:**
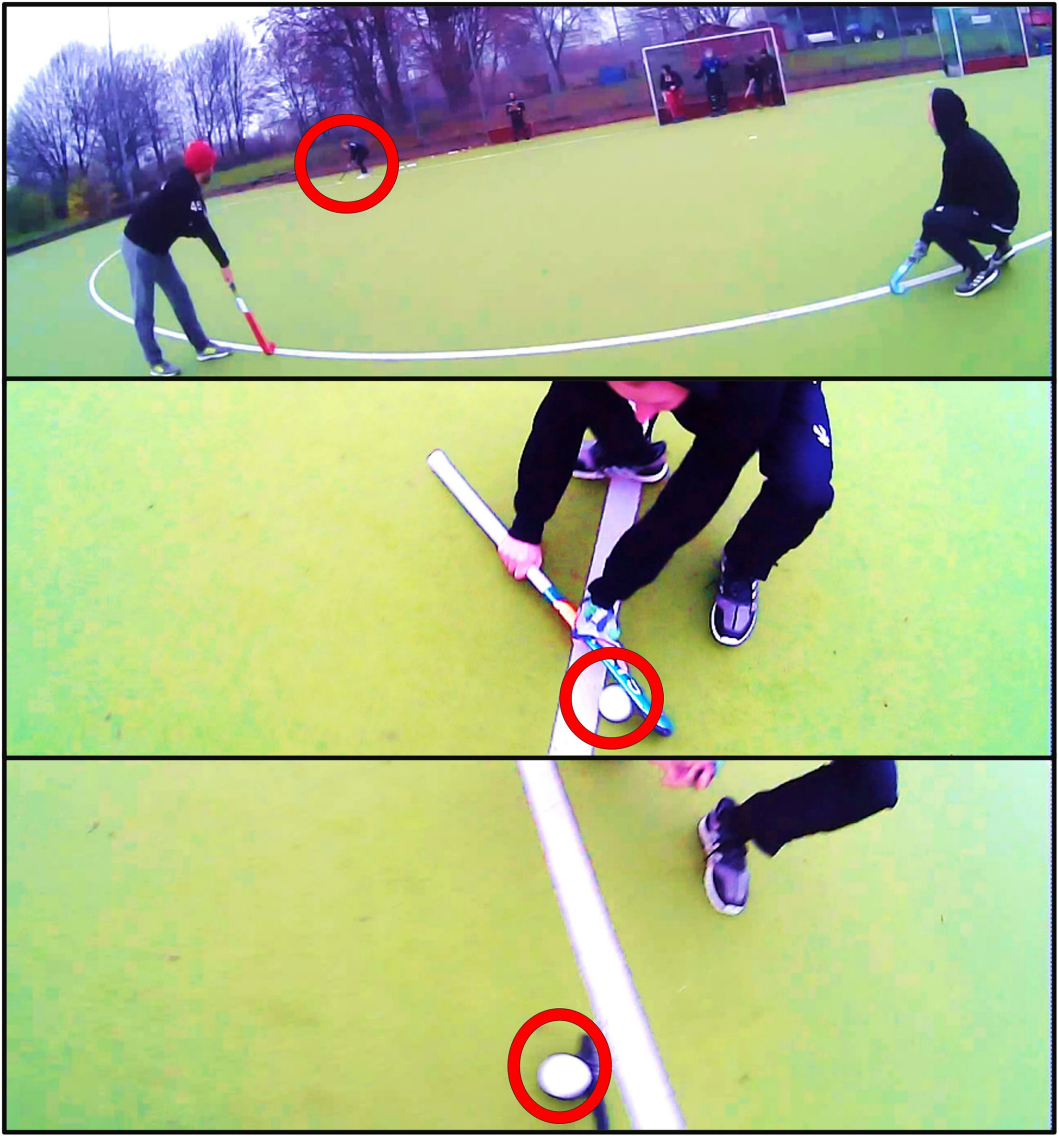
Pictures of a penalty corner attempt from phase 1 (top) to phase 3 (bottom). Phase 1 starts the moment the ball is injected and ends when the ball reaches the stopper's stick. Phase 2 has been defined as the period from the moment the ball leaves the stopper's stick until the striker receives the ball. Phase 3 ended at the moment the pass to the teammate was played or ball was shot.

Next, the video material of the eye-tracking system was calibrated offline in order to monitor the visual foci of the participants during the execution of the penalty corners. A manual frame-by-frame analysis was used to analyze the strikers' gaze behavior using the software Kinovea (Version 0.8.15; for a similar procedure, see Fasold et al., 2018). We only focused on the analysis of gaze duration and left out analyses of other gaze parameters (but see Di Nocera et al., 2007; Noël and van Der Kamp, 2012). Thereby, as common in velocity algorithms (Holmqvist et al., 2011) we included smooth pursuits of moving areas of interest, for example the ball, in our count of gaze durations (cf. Dicks et al., 2010; Aksum et al., 2020). Only if an area of interest was focused for 4 consecutive frames (120 ms) this was counted as gaze at a certain location. A second rater rated 10% of the trials in order to gain information on the reliability of the first rater's work. Cohen's Kappa was found to be 0.77 (“substantial agreement,” cf. Landis and Koch, 1977). In order to be able to draw conclusions about the gaze and decision-making behavior of the participants, areas of interest were defined for the penalty corners, which could be observed by the striker during the shooting process. The ball, the injector, the stopper, the goal area, the shooting players in 90° variation, and the deflection variation were defined as such areas of interest. The starting point of a scene was defined as the moment when the ball is injected, and the end of the shooting process was defined as the moment when the ball has been passed or shot directly by the striker. For a more detailed analysis of the scenes and also to allow testing whether differences between condition occur mainly during the initial phase of a penalty corner or not, the sequence of a penalty corner was divided into three phases: Phase 1 is the period starting from the moment the ball is injected (at this point the defense is allowed to move) until the stopper's stick contact. Phase 2 has been marked as the period from the moment the ball leaves the stopper's stick until the striker receives the ball. Phase 3 ended at the moment the pass to the teammate was played or ball was shot (see Figure 4). Finally, data was analyzed using a 2 (condition: defense-dependent, defense-independent) × 3 (phase: phase 1, phase 2, phase 3) MANOVA with repeated measures for both factors and gaze duration at the different areas of interest (ball, injector, stopper, goal area, deflecting player, 90° player) as dependent variables. Subsequently, data collected for the “defense-dependent” condition was analyzed by means of a 2 (decision quality: optimal; less appropriate) × 3 (phase: phase 1; phase 2; phase 3) × 2 (variation: deflection; 90°) MANOVA^3^ with repeated measures for all factors and again gaze duration at the different areas of interest (ball, injector, stopper, goal area, deflecting player, 90° player) as dependent variables. Assumptions for calculating a MANOVA were tested and in case of any violations of sphericity, Greenhouse-Geisser correction was applied. We followed both MANOVAs up with (univariate) ANOVAs in order to relate significant multivariate effects to single dependent variables.

### Results

The data of a total of 253 penalty corners could be used for the analyses of gaze. Seven penalty corners could not be included in the data analysis due to technical problems. In total, the recordings consisted of 15,443 frames, of which 11,434 (74.04%) were included in the data analysis, as in 3,999 frames (25.9%), gaze was not detected. From Phase 1, there were 7,497 frames, of which 6,937 frames (92.53%) were included in the analysis. In phase 2, 4,047 frames were recorded, of which 2,426 frames (59.95%) showed no gaze marker. From the last phase, 2,080 frames (53.31%) of 3,902 frames could be considered for data analysis.

#### Differences Between Conditions

Results of the MANOVA showed a main effect of phase, *V* = 1.761; *F*_(10, 42)_ = 30.981, *p* < 0.001, η_*p*_^2^ = 0.881, of condition, *V* = 0.873; *F*_(5, 8)_ = 10.964, *p* = 0.002, η_*p*_^2^ = 0.873, and an interaction of phase and condition, *V* = 0.955; *F*_(10, 42)_ = 3.835, *p* = 0.001, η_*p*_^2^ = 0.477, on the participants' distribution of gaze on the different areas of interest.

Results of subsequent univariate analyses are shown in Table 1. Participants looked longer at the ball and injector (*M* = 51.971, *SE* = 4.494, 95% *CI* [42.179; 61.762]; *M* = 5.832, *SE* = 0.743, 95% *CI* [4.212; 7.452]) in the cases where a defense-independent strategy was adopted than in cases of a defense-dependent strategy (*M* = 40.294, *SE* = 3.730, 95% *CI* [32.166; 48.422]; *M* = 3.493, *SE* = 0.607, 95% *CI* [2.171; 4.816]). The opposite was true with regard to gaze durations on the goal area (defense-dependent: *M* = 13.940, *SE* = 1.444, 95% *CI* [10.793; 17.087]; defense-independent: *M* = 3.722, *SE* = 0.859, 95% *CI* [1.850; 5.595]).

**Table 1:** Summary of univariate analyses of gaze durations in defense-dependent and independent strategy for the three phases of the penalty corner process.

**Effect**	**Dependent variable**	**F (dfs)**	***p***	**η_p_^2^**
**Condition**	**Ball**	**14.035 (1, 12)**	**0.003**	**0.539**
	**Injector**	**17.923 (1, 12)**	** <0.001**	**0.599**
	Stopper	0.131 (1, 12)	0.724	0.011
	**Goal area**	**43.772 (1, 12)**	** <0.001**	**0.785**
	Deflecting player	0.99 (1, 12)	0.337	0.077
**Phase**	**Ball**	**9.898 (2, 24)**	**0.001**	**0.452**
	**Injector**	**41.773 (1.039, 12.473)**	** <0.001**	**0.777**
	**Stopper**	**36.539 (2, 24)**	** <0.001**	**0.753**
	**Goal area**	**59.372 (1.125, 13.497)**	** <0.001**	**0.832**
	Deflecting player	0.99 (2, 24)	0.383	0.077
Condition*Phase	**Ball**	**13.660 (2, 24)**	** <0.001**	**0.532**
	**Injector**	**10.736 (1.312, 15.740)**	**0.003**	**0.472**
	Stopper	2.022 (1.271, 15,256)	0.154	0.144
	**Goal area**	**52.095 (1.066, 12.786)**	** <0.001**	**0.813**
	Deflecting player	0.99 (2, 24)	0.337	0.077

*These were used following a MANOVA in order to detect for which dependent variables differences exist. The columns including p values <0.05 are in bold*.

**Figure 5:**
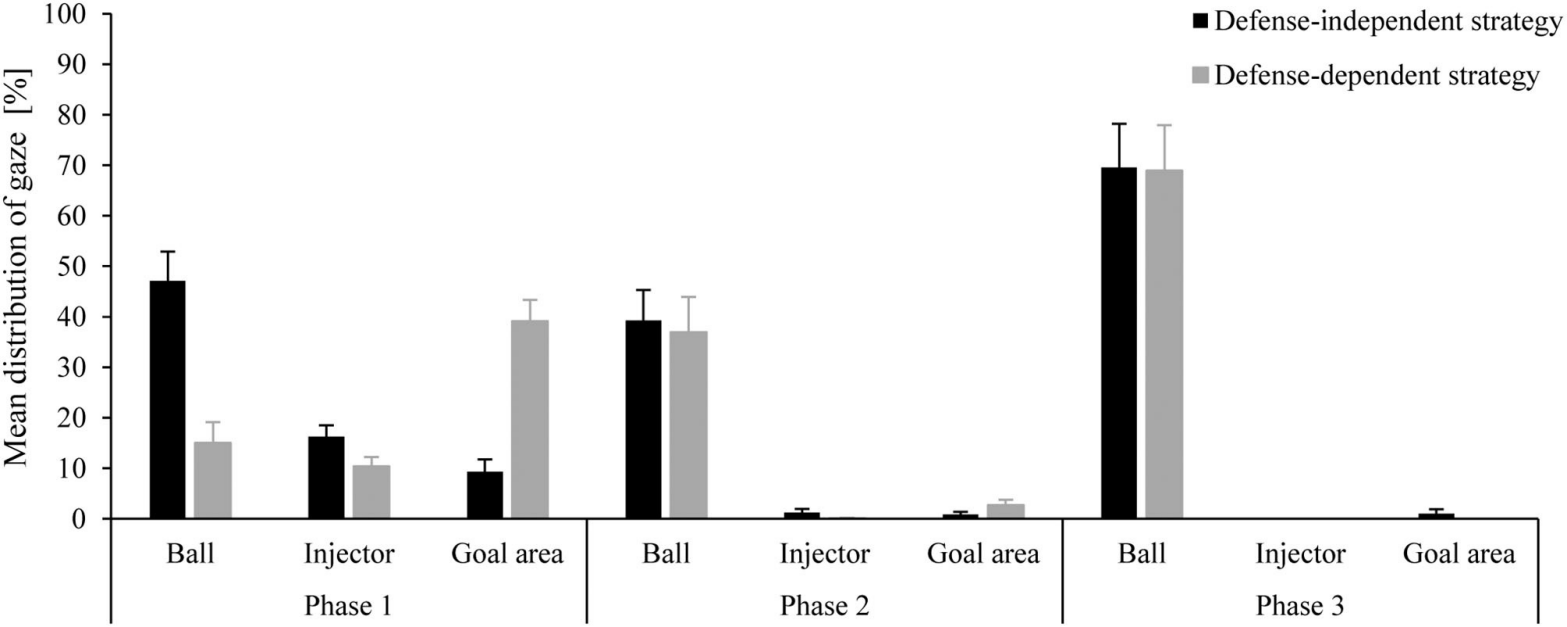
Mean distribution (and SD) of gaze durations at the areas of interest (ball, injector, goal area) for both conditions (defense-independent; defense-dependent) as a function of time (Phase 1, Phase 2; Phase 3) and in relation to the length of a phase.

However, while gaze duration at the ball during the defense-dependent and defense-independent conditions was roughly the same in phase 2 (*M* = 39.267, *SE* = 6.020, 95% *CI* [26.150; 52.384] vs. *M* = 36.977, *SE* = 6.932, 95% *CI* [21.873; 52.082]) and phase 3 (*M* = 69.518, *SE* = 8.696, 95% *CI* [50.571; 88.465] vs. *M* = 68.914, *SE* = 9.023, 95% *CI* [49.254; 88.575]), during phase 1, players focused on the ball longer in the defense-independent compared to the defense-dependent condition (*M* = 47.127, *SE* = 5.785, 95% *CI* [34.523; 59.732] vs. *M* = 14.990, *SE* = 4.137, 95% *CI* [5.976; 24.004]). In phase 1, players looked longer at the injector in cases they already knew how to carry out the penalty corner compared to the defense-dependent condition (*M* = 16.237, *SE* = 2.258, 95% *CI* [11.316; 21.157] vs. *M* = 10.385, *SE* = 1.854, 95% *CI* [6.346; 14.424]). However, this difference between conditions got smaller during phase 2 (*M* = 1.260, *SE* = 0.702, 95% *CI* [−0.271; 2.790] vs. *M* = 0.095, *SE*= 0.095, 95% *CI* [−0.112; 0.302]) and during phase 3, players in both the situations never looked at the injector. The gaze durations at the goal area during phase 1 (*M* = 39.123, *SE* = 4.221, 95% *CI* [29.925; 48.320] vs. *M* = 9.276, *SE*= 2.478, 95% *CI* [3,878; 14.674]) and phase 2 (*M* = 2.697, *SE* = 1.044, 95% *CI* [0.424; 4.971] vs. *M* = 0.870, *SE* = 0.490, 95% *CI* [−0 to 197; 1.937]) were longer in the defense-dependent compared to the defense-independent condition. But in phase 3, the goal area was only sparsely looked at in the defense-independent condition (*M* = 1.021, *SE* = 1.044, 95% *CI* [-0.846; 2.888]) (Figure 5).

The scoring rates of both conditions were rather similar. In the defense-dependent condition, the offense scored in 15.87% of the attempts whereas scoring rate was 16.54% in the defense-independent condition.

#### Differences Within Defense-Dependent Condition

In 65.08% of the trials, the striker chose the optimal strategy to counter the defenders' run out. Results of the second MANOVA showed again that gaze differed between the phases of the penalty corner, *V* = 1.556; *F*_(8, 44)_ = 19.296, *p* < 0.001, η_*p*_^2^ = 0.778, but also that gaze was different for penalty corners in which players were able to respond to the behavior of the defense in an optimal compared to a less optimal way, *V* = 0.756; *F*_(4, 9)_ = 6.971, *p* = 0.008, η_*p*_^2^ = 0.756. Furthermore, there was an interaction between both factors, *V* = 1.196; *F*_(8, 44)_ = 8.174, *p* < 0.001, η_*p*_^2^ = 0.598, whereas gaze differences between phases weren't the same for deflection and 90° variations, *V* = 0.595; *F*_(8, 44)_ = 2.327, *p* = 0.035, η_*p*_^2^ = 0.297. Gaze did not differ between both penalty corner variants, though, *V* = 0.421; *F*_(4, 9)_ = 1.634, *p* = 0.248.

Results of subsequent, univariate analyses are shown in Table 2. In the cases where the players' behavior optimally matched the tactical formation of the defense, they spent more time looking at the ball (*M* = 40.429, *SE* = 3.720, 95% *CI* [32.324; 48.534] vs. *M* = 16.187, *SE* = 5.183, 95% *CI* [4.894; 27.481]; *p* < 0.001), the stopper (*M* = 3.463, *SE* = 0.763, 95% *CI* [1.800; 5.127] vs. *M* = 7.597, *SE* = 2.118, 95% *CI* [2.982; 12.212]; *p* = 0.001), and the goal area (*M* = 14.399, *SE* = 1.437, 95% *CI* [11.269; 17.530] vs. *M* = 6.071, *SE* = 1.777, 95% *CI* [2.199; 9.942]; *p* < 0.001).

**Table 2:** Summary of univariate analyses of gaze in defense-dependent strategy as a function of decision quality (optimal vs. less appropriate) and phase of the penalty corner.

**Effect**	**Dependent variable**	**F (dfs)**	***p***	**η_p_^2^**
**Decision quality**	**Ball**	**24.075 (1, 12)**	** <0.001**	**0.667**
	Injector	3.898 (1, 12)	0.072	0.245
	**Stopper**	**20.055 (1, 12)**	**0.001**	**0.626**
	**Goal area**	**25.116 (1, 12)**	** <0.001**	**0.677**
Decision quality*Phase	**Ball**	**9.319 (2, 24)**	**0.001**	**0.437**
	Injector	3.406 (1.007, 12.088)	0.089	0.221
	**Stopper**	**11.828 (2, 24)**	** <0.001**	**0.496**
	**Goal area**	20.216 (1.247, 14.960)	*p* < 0.001	0.628
Variant*Phase	Ball	0.191 (2, 24)	0.828	0.016
	**Injector**	**7.406 (1.034, 12.404)**	**0.017**	**0.382**
	Stopper	3.193 (1.034, 12.432)	0.09	0.21
	Goal area	0.795 (1.238, 14.858)	0.413	0.062

*These were used following a MANOVA in order to detect for which dependent variables differences exist. The columns including p values <0.05 are in bold*.

**Figure 6:**
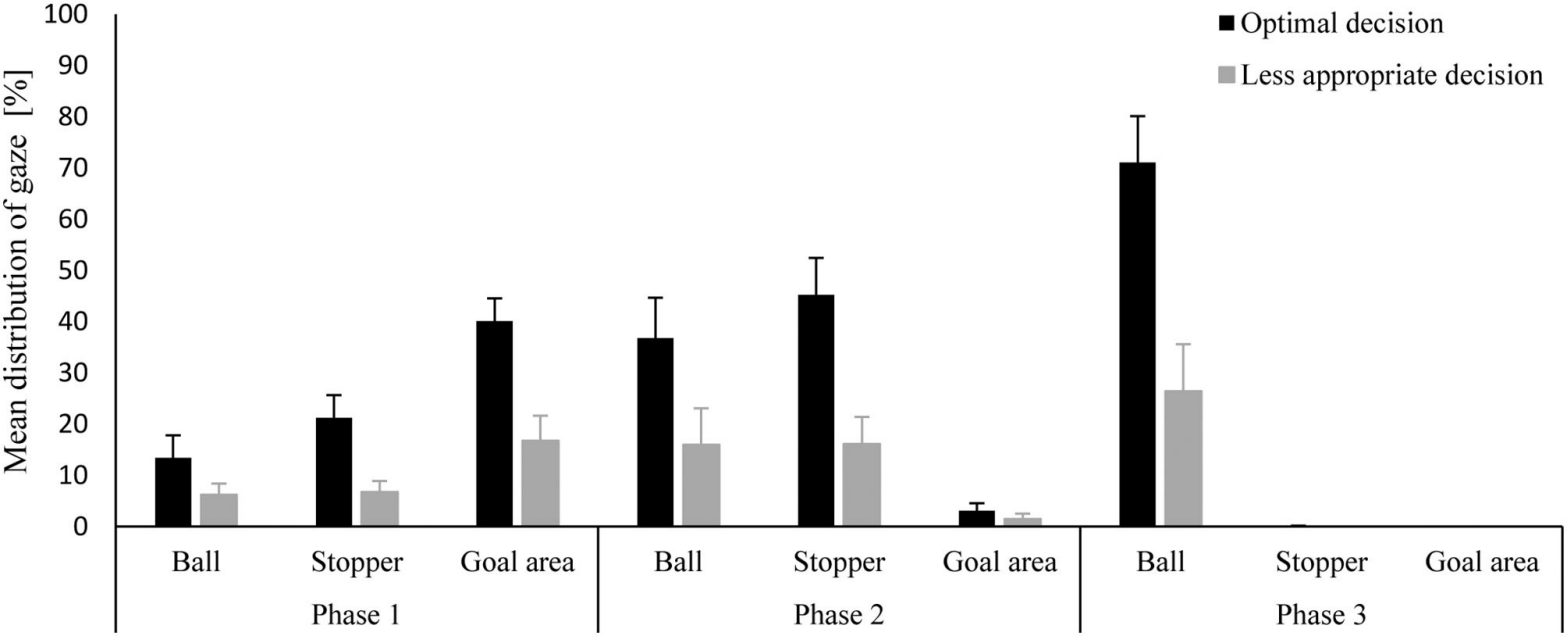
Mean distribution (and SD) of gaze duration at the areas of interest (ball, stopper, goal area) for the evaluation of how well an offensive strategy has matched the run-out of defenders (optimal; less appropriate) as a function of time (Phase 1, Phase 2; Phase 3) and in relation to the length of a phase.

However, these differences based on the appropriateness of the chosen tactical approach, appeared to be inconsistent across the different phases of the penalty corner (Figure 6). In the cases where the variant of the penalty corner matched the defensive formation optimally, players spent more time looking at the ball (optimal: *M* = 13.376, *SE* = 4.411, 95% *CI* [3.765; 22.987] vs. less appropriate: *M* = 6.223, *SE* = 2.173, 95% *CI* [1.490;10.957]), the stopper (optimal: *M* = 21.245, *SE* = 4.429, 95% *CI* [11.596; 30.894] vs. less appropriate: *M* = 6.702, *SE* = 2.179, 95% *CI* [1.955; 11.448]), and the goal area (optimal: *M* = 40.127, *SE* = 4.407, 95% *CI* [30.525; 49.728] vs. less appropriate: *M* = 16.717, *SE* = 4.924, 95% *CI* [5.988; 27.445]) in phase 1 than in the cases where the chosen play design was less appropriate.

Regarding gaze spent at the ball, this difference got bigger in phase 2 (optimal: *M* = 36.817, *SE* = 7.848, 95% *CI* [19.718; 53.915] vs. less appropriate: *M* = 15.962, *SE* = 7.116, 95% *CI* [0.457; 31.466]), and was biggest in phase 3 (optimal: *M* = 71.095, *SE* = 9.040, 95% *CI* [51.398; 90.793] vs. less appropriate: *M* = 26.377, *SE* = 9.221, 95% *CI* [6.286; 46.468]). This pattern was different for other areas of interest. Although, the differences in gaze duration for the stopper were biggest in phase 2 (optimal: *M* = 45.260, *SE* = 7.177, 95% *CI* [29.622; 60.897] vs. less appropriate: *M* = 16.090, *SE* = 5.295, 95% *CI* [4.552; 27.627]), players hardly looked at the stopper in the third phase. Differences in relation to gaze durations for the goal area disappeared after phase 1 (after which the goal area was hardly focused on at all).

### Discussion

In Study 2, the gaze behavior of strikers was recorded in defense-dependent and -independent conditions. The results show that both strategies differ in important aspects of their gaze behavior, mainly in phase 1. This is in line with the descriptions of gaze behavior of players in Study 1 which was unexpected because the self-reports on gaze are not always reliable. In this case, however, players' temporal and spatial description of acquiring information on defenders' behavior matched well with what we actually observed in Study 2. This pattern of gaze allowed players to choose the strategy that matched the run-out of defenders optimally in almost two out of three penalty corners. Players in the defense-dependent condition appear to spend more time looking at the goal area at the expense of gaze durations on the ball and injector (until the ball has reached the stopper and the strikers have to initiate their own actions). In fact, the ball was fixated considerably less (~15%) compared to the defense-independent condition (~47%). In the defense-dependent condition, on the other hand, the goal area was fixated longer (~39%) than in the defense-independent condition (~9%). That is, as hypothesized players in the defense-dependent condition had to manage two sources of information, one providing information on the defense (i.e., which variation to choose) and one providing information that seemed necessary for more optimal execution of their following actions (shot, pass). In contrast, players in the defense-independent strategy knew the offensive variation already before the injection of the ball what allowed them to mainly focus on the ball (and stopper) throughout the initial course of the penalty corner attempt. Rothkopf et al. (2007) emphasized that areas of high interest are focused on at the beginning of a phase or a sequence. But probably because the players in the defense-dependent condition needed sufficient time to not only perceive the pattern of defenders' run-out behavior, but also to react to it in an appropriate way, differences between conditions mainly vanished after phase 1. However, during the penalty corner process, strategies did not lead to different patterns of gaze because the focus shifted predominantly on movement execution.

In phase 2, this involves gaze at the stopper who indicates the striker where to pick up the ball, whereas in phase 3, gaze was almost exclusively directed at the ball to guide the execution of strikers' actions. The latter is also supported by a study by Kurz et al. (2018), which showed that focusing the ball is important for a good technical execution. Similar to the current interaction finding Noël and van Der Kamp (2012) revealed that penalty takers paid more visual attention to the goalkeeper in the beginning of their run-up when they were asked to employ a keeper-dependent strategy. Importantly, they also stopped collecting information on the goalkeeper's behavior (jump direction, movement onset of dive) before foot-to-ball contact during their run-up because it would not have been possible to consider very late sources of information on the goalkeeper and successfully react to them anyway. Taken together, the current results indicate that participants indeed tried to react to the defense using adapted gaze strategies to decide on early parts of the defenders run-out behavior (cf. Roth et al., 2007). However, the current results also show that it probably will not be possible to take later aspects of defenders' run-out into account.

Furthermore, the differentiation between gaze behaviors when decision making was considered optimal or less appropriate during the defense-dependent condition, points to the importance of three areas of interest for adaptive decision making: the goal area, the stopper, and the ball. When choosing the optimal variant to counter defenders' behavior, the strikers spent more time looking at each of these areas. Probably, the longer gaze times on the ball and stopper, mainly in the second and third phase respectively, are a consequence of having identified the defenders' strategy in time (in phase 1) after which they can solely focus on the execution of their own actions. In case they have more problems recognizing defenders' strategies, they probably still have to focus on other potentially informative areas before shifting their focus to guidance of their own movements. Spending sufficient time on the goal area, though, seems to be most the important factor during phase 1 to recognize the defenders' behavior correctly. If the strikers chose the optimal strategy, they would spend more than twice as much time looking at the goal area than in the cases where they chose the less appropriate option.

In this current study, participants almost never spent time looking in between areas of interest. This is probably related to the fact that some of the areas of interest are very far apart and also a consequence of the decision to not differentiate between sources of information in the goal area. It seems likely that within this area of interest, participants made use of peripheral vision to perceive several defenders, the goalkeeper, and the target area in the goal, at the same time (cf. Hüttermann and Memmert, 2017). However, given the relatively long distance between the striker and the goal, the short distance between defenders and the fact that the goal and the goalkeeper were right behind them, it seemed impossible to reliably differentiate between gazes to these sources of information. It remains a question for future research, though, to examine the extent to which strikers make use of peripheral vision, especially during defense-dependent strategy where the need to perceive different sources of information seems stronger (cf. Hüttermann et al., 2014).

Furthermore, the strikers were not trained to employ defense-dependent strategy though. That is why it seems reasonable that gaze behavior in this condition would potentially look somewhat different after players/teams have gathered more experience with this strategy. However, despite this fact, the strikers were able to choose an optimal strategy in 65.08% of the cases. This strongly points to adaptive offensive play calling (i.e., providing offenses with at least two variants of which they can choose based on how the defenders run out) as a promising future development to improve penalty corner success. This is supported by the players' self-evaluation provided in Study 1. Furthermore, goal scoring rate was similar in both conditions, but comparisons of percentages of goals scores seems rather problematic. First, the focus of the current study was mainly on gaze behavior and decision making of the striker. However, goal scoring does not only depend on his/her decision making but also on the perception and performance of his/her teammates (especially in case he/she opts to pass and not to shoot directly). Second, goal scoring does not only depend the defense tactical approach but also on other aspects of the players' behavior and performance (cf. Vinson et al., 2013). For instance, a similar shot on the goal will sometimes result in a goal, but sometimes be saved by the goalkeeper. Finally, as stated above, the strikers and also the other offensive players were not trained to adapt their behavior during a penalty corner. That is, goal scoring rate would probably increase after proper training sessions.

## General Discussion

In recent years, various studies have dealt with different gaze strategies whose application is meant to benefit players' decision making and performance in sports games (e.g., Wilson et al., 2015). However, patterns of gaze behavior likely differ between different sports and probably also within different situations/tasks within one sport (Cañal-Bruland and Mann, 2015). This is so because transfer of knowledge in one sport (e.g., gaze behavior in soccer penalty kicks, e.g., Wilson et al., 2009) is not easy and therefore, cannot replace research in other sports, such as field hockey, that have received less attention by sport scientists/sport psychologists in the past. However, the general principles and observations from one sport can indeed help to improve or better understand decision-making behavior in another sport. In the current paper, we tried to scrutinize if and to what extent can strikers in hockey penalty corners also consider the actions of their opponents (as e.g., in soccer penalty kicks, cf. van der Kamp, 2006, 2011). We asked how far a reaction (to an opponent) is be more effective than a self-initiated, already planned action. To this end, previous research on the relationship between action and reaction has mainly focused on movement times (e.g., Welchman et al., 2010). Those findings indicate that reactive movements are usually faster than self-initiated movements (Pinto et al., 2011) and that this holds across different levels of within-task expertise (Martinez de Quel and Bennett, 2014). However, there also seem to be other benefits of choosing to react to an opponents' behavior (cf. Noël et al., 2021). It allows to ultimately choose a strategy that is more promising because decisions are based on more (reliable) information of the opponents' behavior. In contrast, it causes extra problems like time constraints, the need to synchronize more complex processes as a team, and additional demands on gaze behavior, too.

The present study was focused on the investigation of the gaze behavior and decision making of experienced field hockey players during penalty corners. We were interested in how far the offensive players can adapt their strategy to the run-out behavior of the defenders, thus in how far it is reasonable to base their own actions on the perceptions of the opponents' behavior. In Study 1, a questionnaire was used with the goal of obtaining subjective experiences with and opinions on adaptive offensive behavior during penalty corners and its associated gaze behavior mainly in order to subsequently examine gaze behavior of defense-dependent and -independent strategy within the scope of a field study (Study 2). That was necessary because of the explorative nature of the current research and missing information on many basic relationships in this context.

Both the studies together show that adapting to the behavior of the defenders seems possible and is considered a promising future development by most players. Furthermore, a look at the decision-making performance of one of the players with experience as striker in the German senior national team illustrates that especially high-class players after more intensive training are very well capable of reacting to the defenders' run-up in the first phase of the penalty corner. This particular participant always chose the optimal tactic to counter the approach of the defense.

However, it remains to be seen in what way adaptive behavior during penalty corners can be trained because rapid reactions to the defenders run-out do not only afford good decision making by an individual but also communication between offensive players and coordination of their gaze behavior and movements (cf. Fasold et al., 2018, 2021). That is, after strikers have learnt/established a certain pattern of gaze behavior over the course of several training sessions, they are probably able to focus on the right place at the right time (cf. Magill, 1998) and know the more informative areas enabling good decision making (Abernethy, 2001). But implementing of clear arrangements concerning the routes and positions of the other offensive players and how they get informed on the strikers' final decision seems to be al longer learning process. Furthermore, it certainly requires very good technical skills of all attackers to allow for error-free employment of defense-dependent strategy.

Taken together, the current results point to the benefits of employing a defense-dependent strategy (or in more general terms: reacting instead of acting completely self-planed) at least from time to time also to keep defenses uncertain about which variations they should expect. However, employment of such a strategy seemingly requires intense training and a certain skill set among the offensive players allowing them to rapidly change and coordinate their behavior. Furthermore, on a more strategical level, play designers have to determine out of which more specific variations strikers should chose while observing the run-out of defenders. That is, the current study can be considered a first step toward implementing adaptive decision making by the offense, but there is much work left for coaches, players, and researchers to find out under which circumstances defense-dependent strategies work best. For example, it remains to be investigated for future scientific work to what extent the analysis of other parameters of gaze behavior can be used. In this context, it would also be interesting to analyze to what extent the current results can be replicated (if for example it is made use of a dispersion-based algorithm to identify fixations, see e.g., Blignaut and Beelders, 2009) or to what extent other gaze data can support the current results.

Nevertheless, adaptation to the defenders' formation and behavior during game play is also found in other sports as American Football when, for example, a receiver modifies his/her route according to previous instructions based on his interpretation of the defense strategy. Though learning how to adapt during penalty corners appears relatively extensive, employing defense-dependent strategies seems very well implementable. This appears to also be the case in other sports in which a player or a team has to decide between self-initiated actions and waiting for an action of the opponent in order to choose a reaction that matches the opponents' behavior well.

## Data Availability Statement

The original contributions presented in the study are included in the article/Supplementary Material, further inquiries can be directed to the corresponding author/s.

## Ethics Statement

The studies involving human participants were reviewed and approved by German Sport University Cologne. The patients/participants provided their written informed consent to participate in this study.

## Author Contributions

SK, BN, and FF developed the study concept and contributed to the design. AS and LH collected the data. SK and BN wrote the first draft of the manuscript. All authors helped to edit and revise the manuscript and approved the final submitted version of the manuscript.

## Conflict of Interest

The authors declare that the research was conducted in the absence of any commercial or financial relationships that could be construed as a potential conflict of interest.

## Publisher's Note

All claims expressed in this article are solely those of the authors and do not necessarily represent those of their affiliated organizations, or those of the publisher, the editors and the reviewers. Any product that may be evaluated in this article, or claim that may be made by its manufacturer, is not guaranteed or endorsed by the publisher.

## Footnotes

^1^For depictions of the tactical approaches on offense and defense see Supplementary Figures 1–4.

^2^The German questionnaire can be found in the Supplementary Material.

^3^Mean values that were submitted to both MANOVAs can be found in the Supplementary Material.
