# Supplementary material for: Adaptive Gaze Behavior and Decision Making of Penalty Corner Strikers in Field Hockey
Source: Front Psychol. 2021 Aug 2;12:674511. doi: 10.3389/fpsyg.2021.674511 (PMC8366230; doi:10.3389/fpsyg.2021.674511)
Supplement: Supplementary file 1 [file Data_Sheet_1.PDF]

Supplemental Material

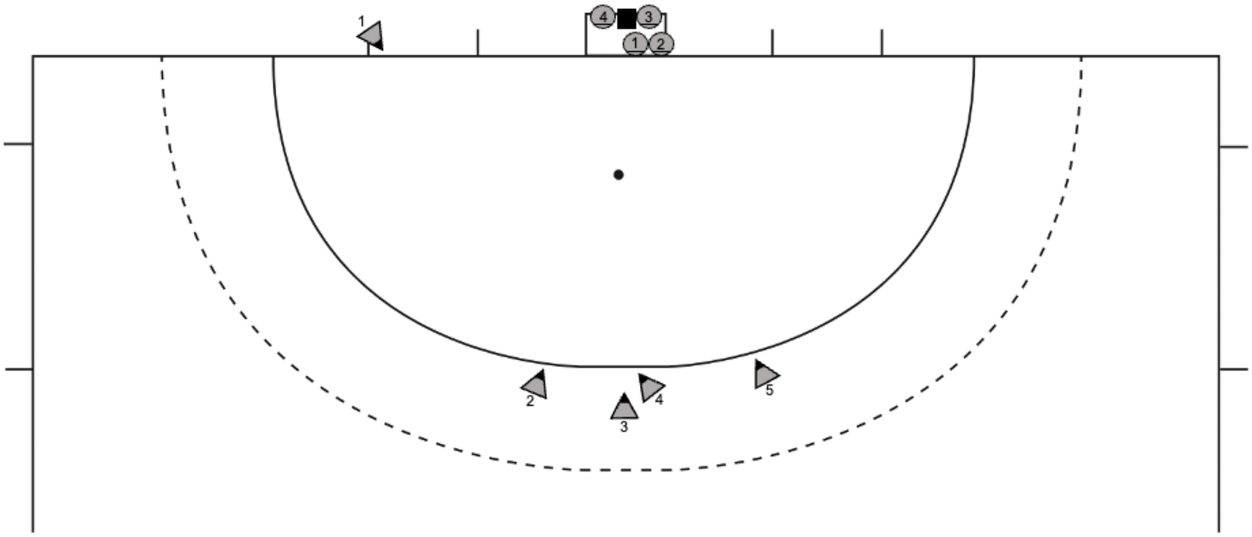

Figure S1. Basic formation. (Triangle 1: injector, triangle 2: 90°-player, triangle 3: striker, triangle 4: stopper, triangle 5: deflection-player; circles: defense; black box: goalkeeper)

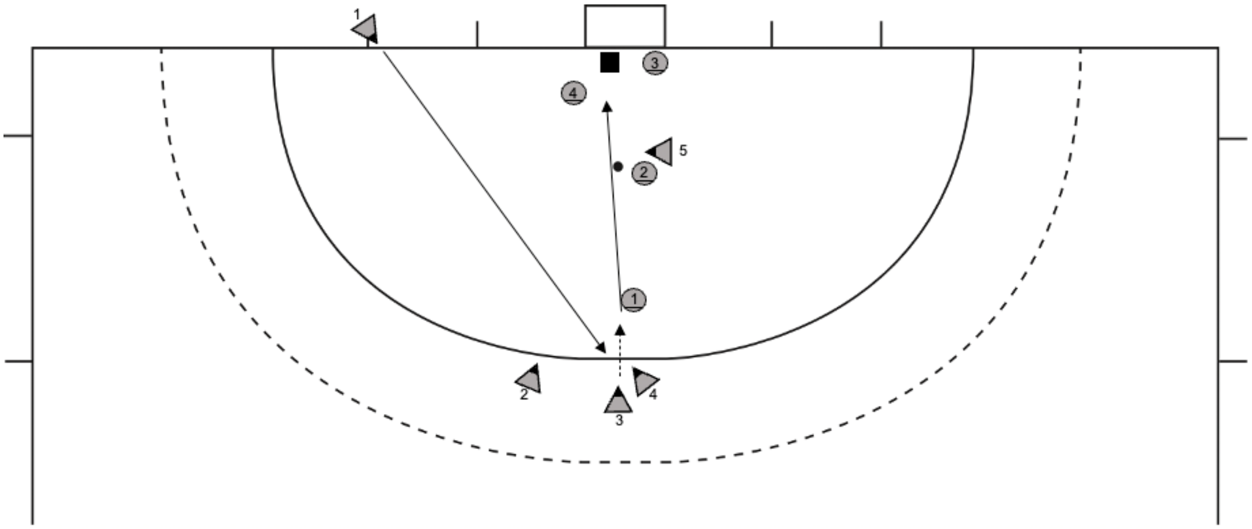

Figure S2. 90° variation and 3:1. Offense: The striker fakes a shot attempt and passes the ball to the player to his left who shoots at the goal. Defense: #1 tries to block a shot, #2 tries to defend deflection variations, #3 defends the right goal side, #4 defends the space in front of the keeper and possible rebounds.

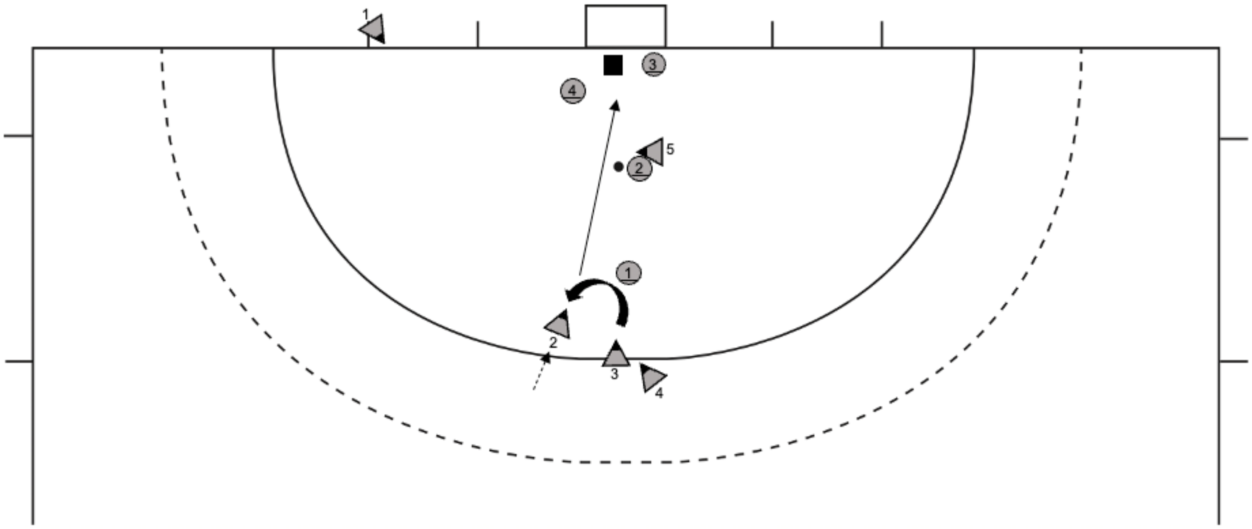

Figure S3. Drag-flick and 3:1. Offense: The injector passes the ball to the stopper who stops the ball outside the circle. The striker drag-flicks at the goal while stepping into the circle. Defense: #1 tries to block a shot, #2 tries to defend deflection variations, #3 defends the right goal side, #4 defends the space in front of the keeper and possible rebounds.

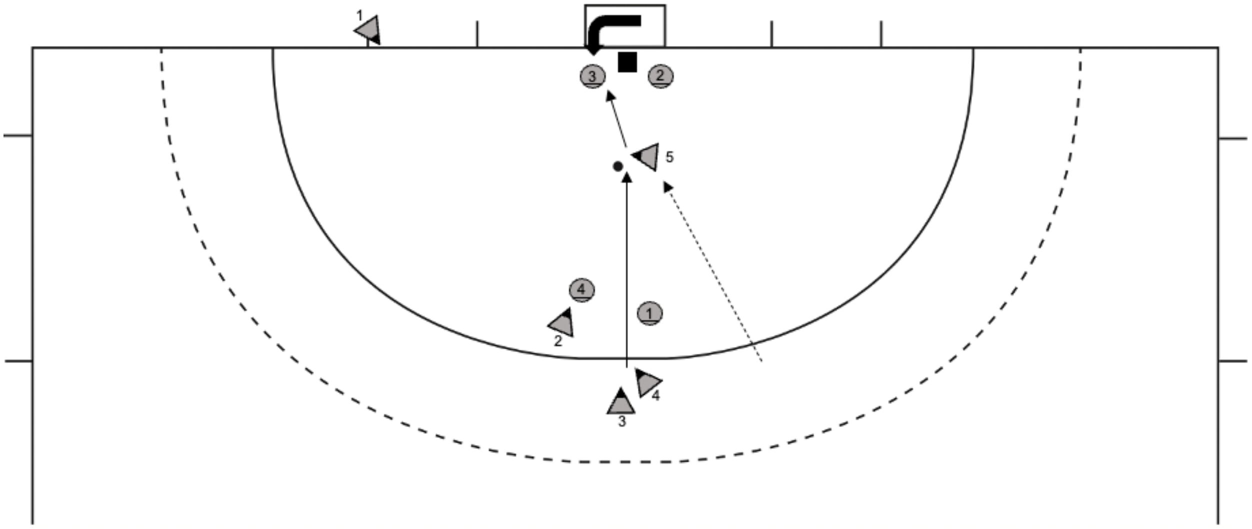

Figure S4. Deflection variation and 2:2. Offense: The striker passes the ball to the player positioned close to the penalty spot who deflects the shot at the goal. Defense: #1 tries to block a shot; #4 tries to defend 90°-variations, #3 defends on the left side, #2 stays on the right side.
