# Supplementary material for: Adaptive Gaze Behavior and Decision Making of Penalty Corner Strikers in Field Hockey
Source: Front Psychol. 2021 Aug 2;12:674511. doi: 10.3389/fpsyg.2021.674511 (PMC8366230; doi:10.3389/fpsyg.2021.674511)
Supplement: Supplementary file 2 [file Data_Sheet_2.PDF]

Fragebogen (German Questionnaire Field Hockey)

Demographische Angaben

Alter:
…………………………………………………………………………………………….

Geschlecht: m __ w__ d__

Grundlegendes:

1. Welche ist die höchste Liga, in der Sie aktiv Feldhockey gespielt haben?

□ 1. Bundesliga
□ 2. Bundesliga
□ Regionalliga
□ Sonstige Angaben:
……………………………………………………………………………………………

2. Wie lange haben Sie in dieser Liga gespielt?

□ < 1 Jahr
□ 1 - 5 Jahre
□ 5 - 10 Jahre
□ > 10 Jahre

3. Welche Priorität nehmen Sie als Eckenschütze/Eckenschützin auf der Schussposition in ihrer Mannschaft ein?

□ 1
□ 2
□ 3
□ Sonstige Angaben:
……………………………………………………………………………………………

Vorbereitung:

4. **Führen Sie neben Ihren Teamtrainingseinheiten auch separates Strafeckentraining durch?**

□ Ja □ Nein, direkt weiter zu Frage 6.

a. *Wie viele Einheiten haben Sie davon in einer Trainingswoche?*

□ 1
□ 2
□ 3
□ > 3

b. *Wie lange dauert dieses spezifische Strafeckentraining?*

□ 15-30 Minuten
□ 20-45 Minuten
□ 45-60 Minuten
□ > 60 Minuten

5. **In wie vielen Einheiten in der Trainingswoche trainieren Sie Strafecken? Zählen Sie dazu bitte auch separates Strafeckentraining.**

□ 1
□ 2
□ 3
□ > 3

6. **Wie viel Zeit wird pro Trainingseinheit investiert?**

□ < 15 Minuten
□ 15-30 Minuten
□ > 30 Minuten
□ Sonstige Angaben:
………………………………………………………………………………………………

Vorbereitung – Offensive:

7. **Welche Offensiv-Varianten spielen Sie bevorzugt bei Strafecken? Mehrfachnennungen möglich.**

□ Schuss
□ Stecher-Variante
□ Ablage links
□ Ablage rechts
□ Sonstige Angaben:
……………………………………………………………………………………………...

8. Wie bereiten Sie sich auf die gegnerische Defensive vor?

□ Videoanalyse
□ Gezieltes Training
□ Keine Vorbereitung
□ Sonstige Angaben:
…………………………………………………………………………………………..

9. Wann erfolgen Entscheidungen für die Strafecken-Variante (offensiv)? Mehrfachnennungen möglich.

□ Vor dem Spiel
□ In Viertel- und Halbzeitpausen
□ Während des Spiels
□ Kurz vor der Strafecke
□ Während der Strafecke
□ Sonstige Angaben:
…………………………………………………………………………………………..

Vorbereitung – Defensive:

10. Welche Defensiv-Varianten spielen Sie bevorzugt bei Strafecken? Die Ausführung der Varianten ist am Ende des Fragebogens schematisch dargestellt (siehe Abbildungen 1 und 2). Mehrfachnennungen möglich. Falls spezielle Ausführungen der bereits aufgezählten Varianten vorhanden, bitte unter „Sonstige Angaben" kurz erläutern.

□ 3:1 → 3:1
□ 3:1 → 2:2
□ 2:2 → 2:2
□ Sonstige Angaben:
…………………………………………………………………………………………..

11. Wie bereiten Sie sich auf die gegnerische Offensive vor?

□ Videoanalyse
□ Gezieltes Training
□ Keine Vorbereitung
□ Sonstige Angaben:
…………………………………………………………………………………………..

12. Wann erfolgen Entscheidungen für die Strafecken-Variante (defensiv)? Mehrfachnennungen möglich.

□ Vor dem Spiel
□ In Viertel- und Halbzeitpausen
□ Während des Spiels
□ Kurz vor der Strafecke
□ Während der Strafecke
□ Sonstige Angaben:

………………………………………………………………………………………………..

Offensive – Defensive Lösungsmöglichkeiten:

13. Welche Lösungsmöglichkeit würden Sie gegen eine 3:1 Defensive anwenden? Mehrfachnennungen möglich.

□ Schuss
□ Stecher-Variante
□ Ablage links
□ Ablage rechts
□ Sonstige Angaben:

………………………………………………………………………………………………..

14. Welche Lösungsmöglichkeit würden Sie gegen eine 2:2 Defensive anwenden? Mehrfachnennungen möglich.

□ Schuss
□ Stecher-Variante
□ Ablage links
□ Ablage rechts
□ Sonstige Angaben:

………………………………………………………………………………………………..

Zusatz: Falls Sie bei Frage 10 unter „Sonstige Angaben“ eine weitere Variante erwähnt haben, welche Lösungsmöglichkeit würden Sie gegen diese anwenden? Mehrfachnennungen möglich.

□ Schuss
□ Stecher-Variante
□ Ablage links
□ Ablage rechts
□ Sonstige Angaben:

………………………………………………………………………………………………..

Während des Spiels:

15. Haben Sie schon einmal Anweisungen bekommen, wohin Sie bei der Durchführung einer Strafecken-Variante schauen sollen?

□ Ja □ Nein, weiter zur Frage 18.

a. *Wie lautete die Anweisung?*

……

16. In welcher Reihenfolge fixieren Sie Anhaltspunkte während der Strafecken-Variante: Schuss? Bitte nennen und ordnen Sie Ihre Blickpunkte dementsprechend. Mehrfachnennungen möglich.
Mögliche Blickpunkte: RausgeberIn, Ball, StopperIn, Torfrau/-mann, Verteidigung, MitspielerIn der Variante. Falls vorhanden, weitere Blickpunkte hinzufügen. Beispielantwort: RausgeberIn, Ball, StopperIn, Ball.

……

Bitte tragen Sie in die leeren Kästchen die Varianten ein, die Sie bei Frage 7 angekreuzt haben. Falls Sie keine weiteren Varianten spielen, gehen Sie direkt weiter zur Frage 17:

In welcher Reihenfolge fixieren Sie Anhaltspunkte während der Strafecken-Variante:
☐

……

17. Zu welchen Zeitpunkten während einer Strafecke ist Ihrer Meinung nach ein gezieltes Blickverhalten möglich, um die Defensiv-Variante des Gegners zu erkennen? Priorisieren Sie dafür Ihre Wahl bitte mit den Zahlen 1-5 (1 = am besten geeignet; 5 = am wenigsten geeignet).

Vor der Eckenrausgabe: ……………...
Kurz nachdem der Ball rausgegeben wurde: ……………...
Kurz bevor der Ball gestoppt wird: ……………...
Wenn der Ball gestoppt ist: ……………...
Während der Ball geschossen wird: ……………...

18. Aus welchen Gründen haben Sie die Prioritäten 1-3 der Frage 17 festgelegt?

Priorität 1:
……
……
Priorität 2:
……
……
Priorität 3:
……
……

19. Welche Anhaltspunkte würden Sie wählen, um die gegnerische Defensivtaktik zu erkennen? Mehrfachnennungen möglich.

□ Position Torwart
□ Position 1. Welle
□ Position 2. Welle
□ Position LinienspielerInnen
□ Bewegung des ganzen Verteidigungsblocks
□ Sonstige Angaben:

…………………………………………………………………………………………..

20. Können Sie sich vorstellen, dass eine Entscheidung bzw. Änderung einer Eckenvariante aufgrund des Blickverhaltens kurzfristig möglich ist?

□ Ja □ Nein, weiter zur Frage 21.

a. **Davon ausgehend, dass Sie die Eckenvariante aufgrund Ihres Blickverhaltens kurzfristig ändern würden, welche Varianten würden Sie ausgehend vom Schuss spielen können? Mehrfachnennungen möglich.**

□ Stecher-Variante
□ Ablage
□ Sonstige Angaben:

……………………………………………………………………………………..

b. **Welche Voraussetzungen müssten erfüllt sein, damit eine kurzfristige Entscheidung möglich ist?**

………………………………………………………………………………………………

21. Welche Probleme sehen Sie in einer kurzfristigen Entscheidung?

………………………………………………………………………………………….................
…………………………………………………………………………………………………
…………………………………………………………………………………………………
…………………………………………………………………………………………………

Schematische Darstellung der defensiven Strafecken-Varianten:

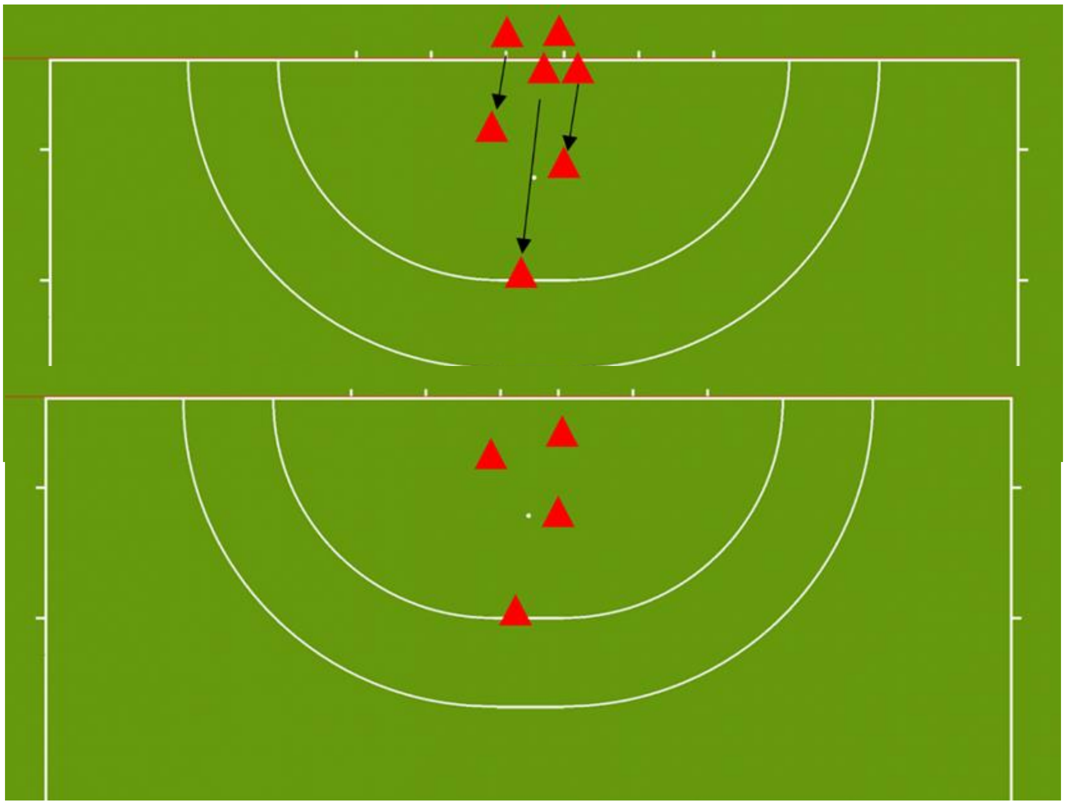

Abb. 1.: Startposition und Laufwege (oben) aus dem 3:1 in das 3:1 (unten).

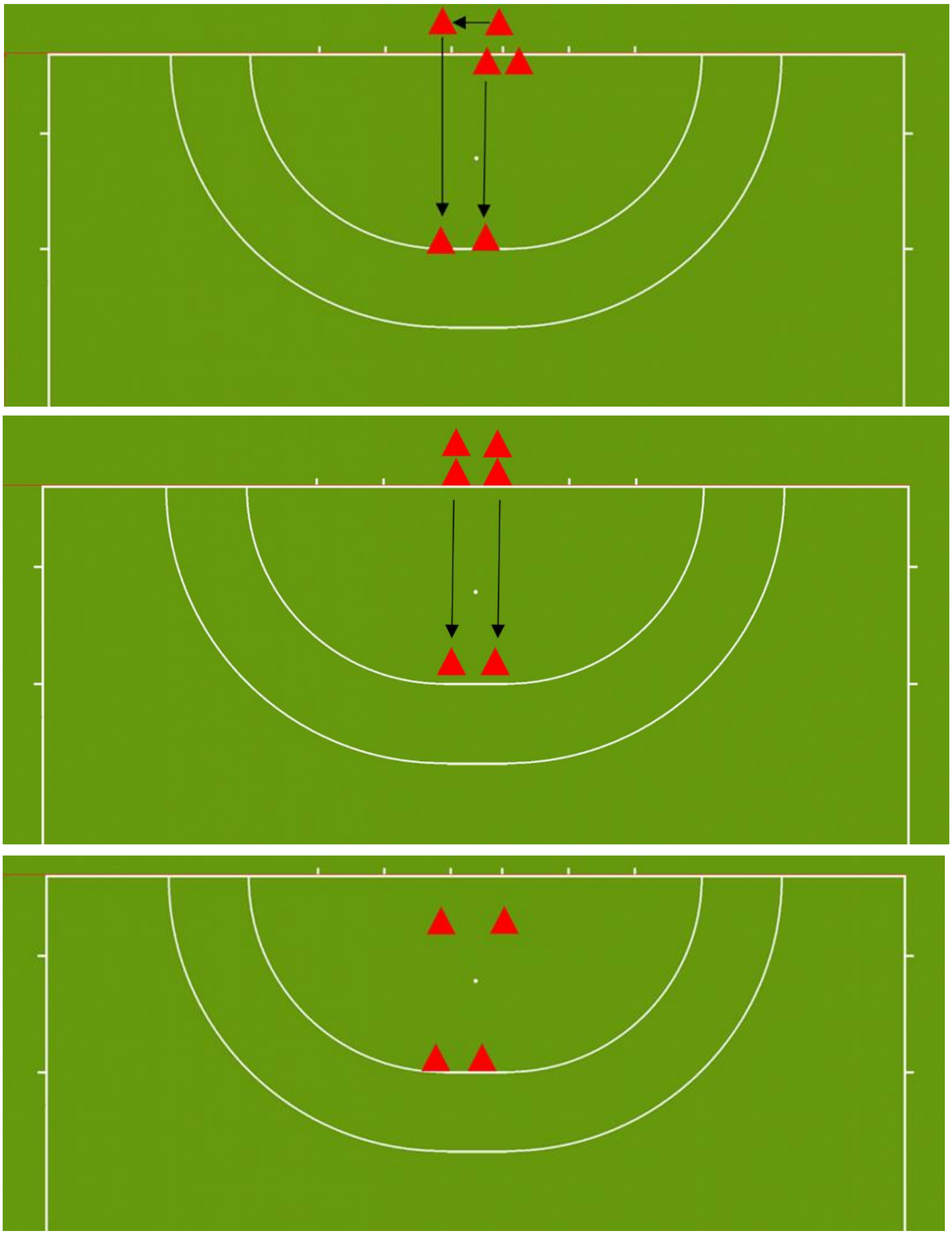

Abb. 2: Startposition und Laufwege aus dem 3:1 (oben) und 2:2 (Mitte) in das 2:2 (unten).
