# Supplementary material for: Adaptive Gaze Behavior and Decision Making of Penalty Corner Strikers in Field Hockey
Source: Front Psychol. 2021 Aug 2;12:674511. doi: 10.3389/fpsyg.2021.674511 (PMC8366230; doi:10.3389/fpsyg.2021.674511)
Supplement: Supplementary file 3 [file Data_Sheet_3.PDF]

Supplemental Material

Table T1. List of offensive strategies chosen by strikers in the defense-dependent strategy as these could not have been kept constant between defense-dependent and defense-independent conditions.

Number of Participant	drag flick	90 degrees	deflection
1	0	5	5
2	3	4	3
3	3	3	4
4	3	4	3
5	5	2	3
6	3	5	2
7	3	3	4
8	1	4	5
9	4	2	4
10	2	4	4
11	3	5	2
12	3	2	5
13	1	4	5
**total**	**32**	**45**	**49**
